# Genome-wide RNAi analysis reveals that simultaneous inhibition of specific mevalonate pathway genes potentiates tumor cell death

**DOI:** 10.18632/oncotarget.4817

**Published:** 2015-08-22

**Authors:** Aleksandra A. Pandyra, Peter J. Mullen, Carolyn A. Goard, Elke Ericson, Piyush Sharma, Manpreet Kalkat, Rosemary Yu, Janice T. Pong, Kevin R. Brown, Traver Hart, Marinella Gebbia, Karl S. Lang, Guri Giaever, Corey Nislow, Jason Moffat, Linda Z. Penn

**Affiliations:** ^1^ Princess Margaret Cancer Centre, Toronto, ON, Canada; ^2^ Department of Medical Biophysics, University of Toronto, Toronto, ON, Canada; ^3^ Donnelly Centre and Banting & Best Department of Medical Research, University of Toronto, Toronto, Canada; ^4^ Institute of Immunology, Medical Faculty, University of Duisburg-Essen, Essen, Germany; ^5^ Now located at AstraZeneca R&D, Mölndal Sweden; ^6^ Now located at Faculty of Pharmaceutical Sciences, University of British Columbia, Vancouver, BC, Canada

**Keywords:** SREBP2, statins, mevalonate pathway, feedback inhibition, tumor metabolism

## Abstract

The mevalonate (MVA) pathway is often dysregulated or overexpressed in many cancers suggesting tumor dependency on this classic metabolic pathway. Statins, which target the rate-limiting enzyme of this pathway, 3-hydroxy-3-methylglutaryl-CoA reductase (HMGCR), are promising agents currently being evaluated in clinical trials for anti-cancer efficacy. To uncover novel targets that potentiate statin-induced apoptosis when knocked down, we carried out a pooled genome-wide short hairpin RNA (shRNA) screen. Genes of the MVA pathway were amongst the top-scoring targets, including sterol regulatory element binding transcription factor 2 (SREBP2), 3-hydroxy-3-methylglutaryl-coenzyme A synthase 1 (HMGCS1) and geranylgeranyl diphosphate synthase 1 (GGPS1). Each gene was independently validated and shown to significantly sensitize A549 cells to statin-induced apoptosis when knocked down. SREBP2 knockdown in lung and breast cancer cells completely abrogated the fluvastatin-induced upregulation of sterol-responsive genes HMGCR and HMGCS1. Knockdown of SREBP2 alone did not affect three-dimensional growth of lung and breast cancer cells, yet in combination with fluvastatin cell growth was disrupted. Taken together, these results show that directly targeting multiple levels of the MVA pathway, including blocking the sterol-feedback loop initiated by statin treatment, is an effective and targetable anti-tumor strategy.

## INTRODUCTION

**Figure 1:**
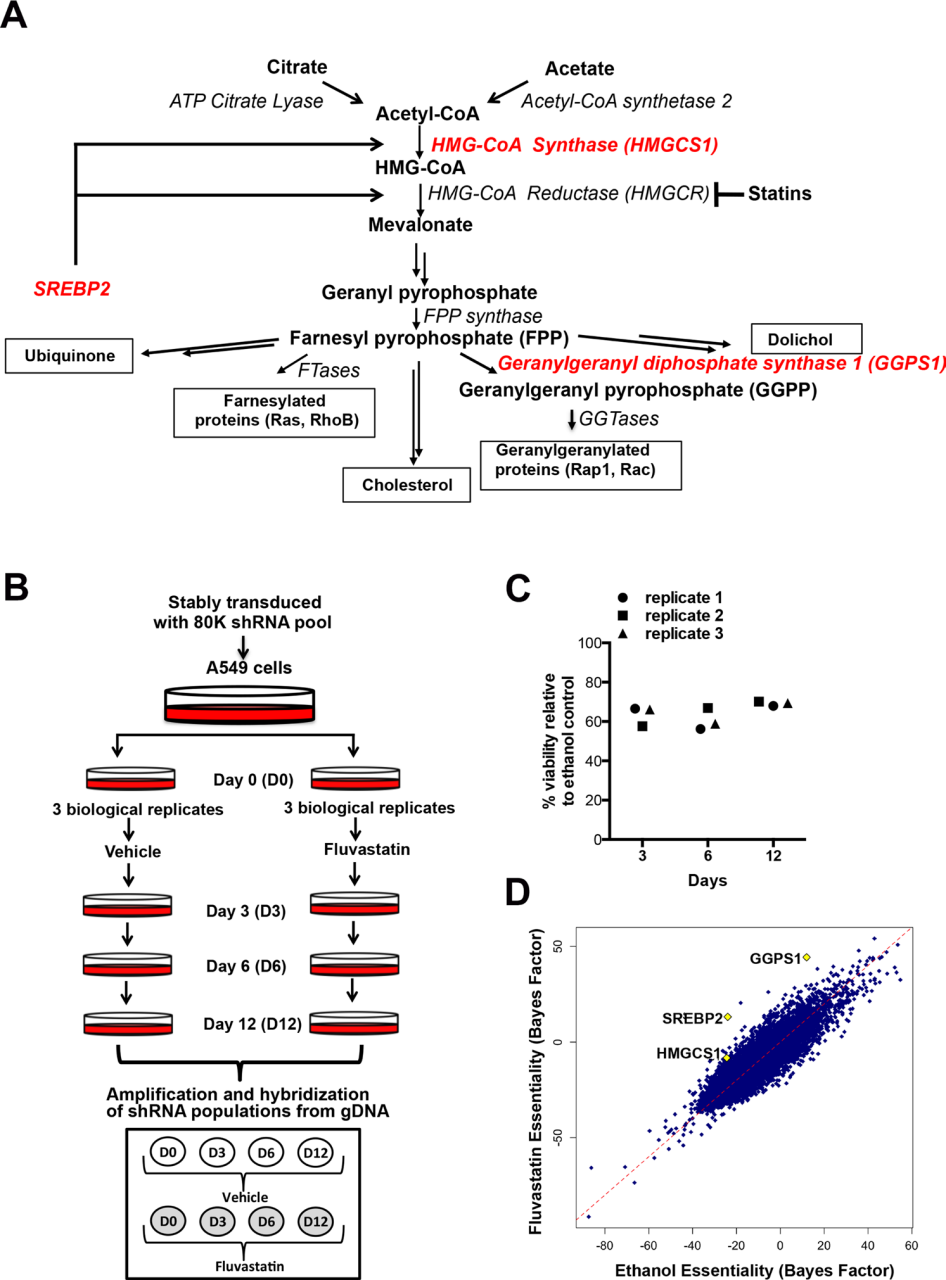
A genome-wide dropout screen uncovers putative shRNAs that potentiate fluvastatin-induced cell death **A.** A simplified schematic of the mevalonate (MVA) pathway. Double arrows represent multiple steps in the pathway. **B.** In the pooled, genome-wide dropout screen A549 cells that had been stably transduced with the library of 80K shRNAs were treated with sublethal doses of fluvastatin (4–5 μM) every 3 days, over 12 days. **C.** Viability as assessed by trypan blue exclusion, relative to control of the fluvastatin-treated replicates. **D.** Significant hits, HMGCS1, GGPS1 and SREBP2, chosen for follow-up are indicated on the scatterplot (diamonds).

Cancer cells undergo drastic metabolic repro-gramming to meet their increased demand for energy and macromolecules. Amongst the metabolic changes occurring in cancer cells is increased *de novo* lipid and cholesterol synthesis through both the fatty acid synthesis and mevalonate (MVA) pathways [1, 2]. The latter not only leads to the production of cholesterol, but also results in important non-sterol end products including farnesyl and geranylgeranyl isoprenoids, dolichol, ubiquinone, and isopentenyladenine (Figure 1A).

In normal cells, the MVA pathway is highly regulated, however, this pathway can be dysregulated in tumor cells by a variety of mechanisms. Tumors frequently have altered metabolism of glucose, glutamine or acetate, which can lead to increased acetyl-CoA, the substrate of the MVA pathway. Solid tumors also often have upregulated ATP citrate lyase and acetyl-CoA synthase 2, both of which produce acetyl-CoA [1–5]. In addition, MVA pathway enzymes can be upregulated by mutant p53 [6] and their elevated expression is associated with poor prognosis and reduced survival in cancer patients [6, 7]. Consistent with this observation, over-expression of the rate-limiting enzyme, 3-hydroxy-3-methylglutaryl-CoA reductase (HMGCR), contributes to oncogenic progression [7]. Furthermore, the restorative feedback response typically found in normal cells is deficient in some tumor cells [8–11]. These multiple levels of MVA pathway dysregulation suggest that cancer cells are particularly dependent on the MVA-derived end products and therefore preferentially sensitive to inhibition of the MVA pathway.

Statins inhibit the MVA pathway and have been successfully used for decades in the control of hypercholesterolemia. Understanding the production and homeostatic regulation of the MVA pathway in normal cells has been instrumental in the development of these effective, well-tolerated cholesterol control agents. Statins inhibit HMGCR leading to the depletion of intracellular cholesterol [12, 13]. This triggers a restorative feedback response mediated by the sterol regulatory element binding protein 2 (SREBP2), which induces the transcription of genes such as HMGCR and low-density lipoprotein receptor (LDLr) [14, 15]. In the liver, this leads to cellular uptake of LDL and the depletion of serum cholesterol levels.

Accumulating epidemiological evidence [16–18] and prospective clinical trials in cancer [19–22] indicate that statins have potential as anti-cancer agents. Evidence suggests that statins can also trigger tumor cells to undergo apoptosis [20, 23–25]. As approved agents, statins can be fast-tracked to impact cancer patient care and targeting the MVA pathway is therefore an important and emerging therapeutic strategy. Cancer therapeutics are not typically used as single agents, but rather delivered as drug cocktails to increase inhibitory activity. To identify novel sensitizers that could combine to maximize the anti-cancer efficacy of statins, we performed a pooled, genome-wide shRNA dropout screen. The A549 cancer cell line was stably transduced with the RNAi Consortium (TRC1) shRNA library [26–28] and exposed to vehicle control or sub-lethal doses of fluvastatin. Genes required for cell survival in the fluvastatin-treated cells were identified using bioinformatics methods as previously described [29]. The top scoring hits included the MVA pathway related genes geranylgeranyl diphosphate synthase 1 (GGPS1), 3-hydroxy-3-methylglutaryl-CoA synthase 1 (HMGCS1), and SREBP2.

Subsequent validation demonstrated that individual knockdown of GGPS1, HMGCS1 or SREBP2 in combination with fluvastatin treatment had anti-proliferative and pro-apoptotic activity. Further characterization revealed that fluvastatin-sensitive lung and breast cancer cells stably expressing shRNAs targeting SREBP2 lost the ability to upregulate HMGCR and HMGCS1 in response to fluvastatin treatment. Furthermore, three-dimensional (3D) growth of these cells knocked down for SREBP2 expression was disrupted following statin exposure, indicating that simultaneously targeting HMGCR and SREBP2 is a promising novel anti-tumor therapeutic strategy. In conclusion, we identified vulnerabilities of the MVA pathway and potential new therapeutic targets that, in combination with statins, can be exploited in the treatment of lung and breast cancer. This paradigm of simultaneous targeting multiple genes within a metabolic pathway is likely instructive for effective targeting of other metabolic tumor vulnerabilities.

## RESULTS

To identify novel sensitizers that potentiate statin-induced anti-proliferative activity and maximize anti-cancer efficacy, we designed a pooled genome-wide dropout shRNA screen (Figure 1B). Lung carcinoma A549 cells were used in this screen as they are relatively insensitive to statin-induced apoptosis. Cells stably expressing shRNA were treated with sublethal (defined as ~30–40% reduction in cell viability, Figure 1C) doses of fluvastatin. Cells sensitive to the combined chemical and genetic perturbation dropped out from the population over the 12 days of the experiment (Figure 1B and 1C). Analysis of the shRNAs remaining in the ethanol control-treated but not fluvastatin-treated cells identified genes that were lethal in cells exposed to fluvastatin. Because A549 cells are relatively statin-insensitive, uncovering statin sensitizers in these cells has the potential to identify vulnerabilities that may be targeted to increase statin anti-cancer activity in tumors otherwise unresponsive to statins. Of 283 significantly under-represented shRNAs following fluvastatin treatment (Supplementary Table 1), a number of genes among the top hits included several related to the MVA pathway (highlighted in red), including HMGCS1, GGPS1 and SREBP2 (Figure 1A and 1D).

HMGCS1 is upstream of HMGCR and synthesizes HMG-CoA, the substrate used by HMGCR in the production of MVA [30]. GGPS1 lies downstream of HMGCR and catalyzes the synthesis of geranylgeranyl pyrophosphate (GGPP), which is used for the post-translational prenylation of many proteins, including small GTP-binding proteins such as the Rho family of proteins integral for cell survival, growth and cytoskeletal organization [31, 32]. SREBP2 plays a critical role in regulating the MVA pathway. In response to intracellular cholesterol depletion, SREBP2 is cleaved and translocates to the nucleus, where it induces the transcription of sterol-responsive genes such as HMGCR and HMGCS1 [14, 15, 33].

**Figure 2:**
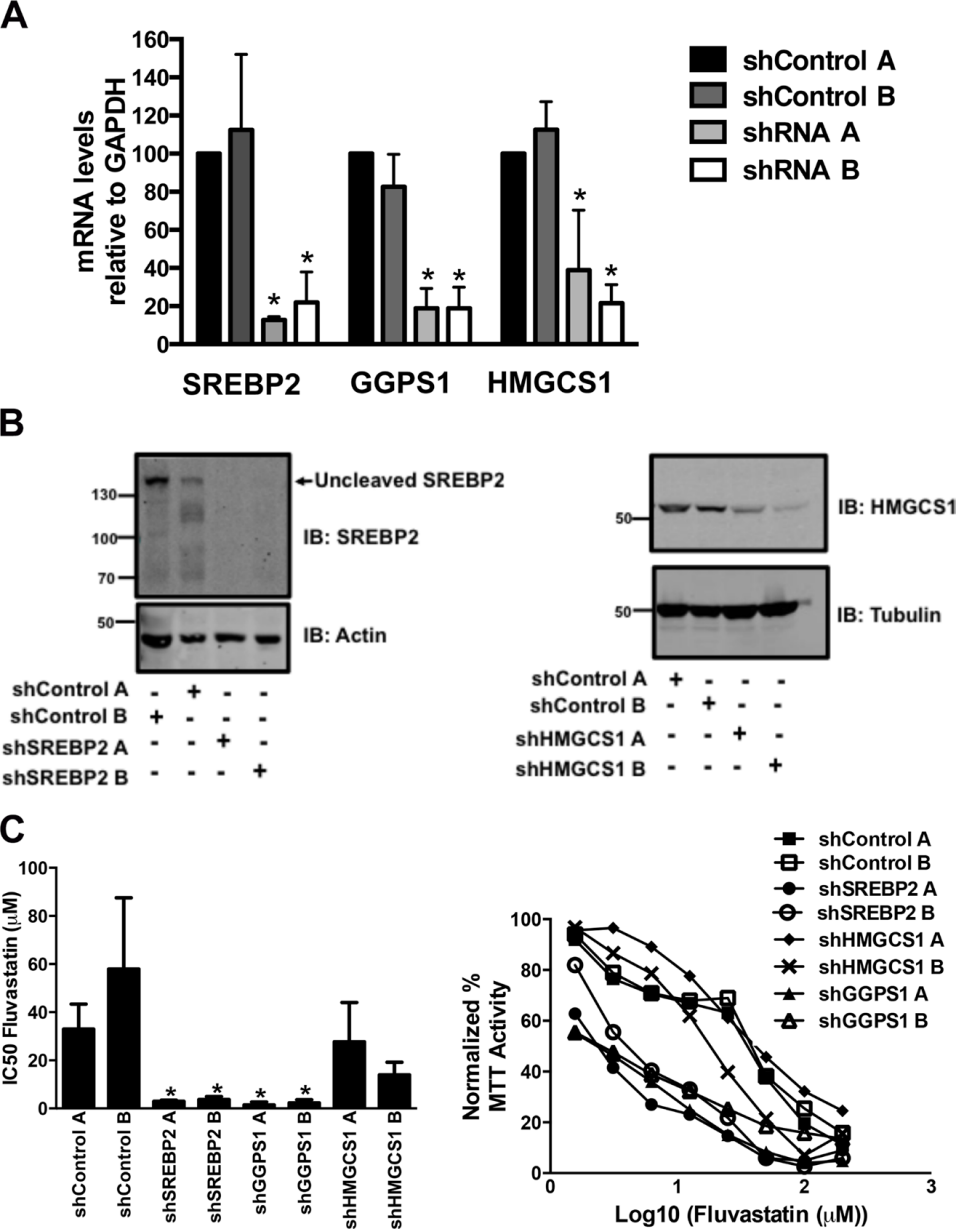
Generation of A549 cell lines stably expressing shRNA constructs targeting genes of the MVA pathway **A-B.** RNA and protein lysates were harvested from asynchronously growing A549 cells that express two independent control shRNAs (shControl A, shControl B) or shRNAs targeting SREBP2, GGPS1 or HMGCS1 (shRNA A, shRNA B). Real-time mRNA EXPRESSION data represents the mean ± SD of three independent experiments. Immunoblots are representative of at least three independent experiments. **C.** Knockdown of SREBP2 and GGPS1 significantly decreased fluvastatin IC50 values. *p < 0.05 (one-way ANOVA with Tukey post-test). Representative dose-response curves assessed using the MTT assay are shown in **C.** (right panel).

We validated potential hits from our shRNA screen by generating A549 cell lines knocked down for HMGCS1, GGPS1 and SREBP2, using two unique shRNA's for each target. These cell lines are referred to as shHMGCS1 A, shHMGCS1 B, shGGPS1 A, shGGPS1 B, shSREBP2 A and shSREBP2 B, respectively. Control cell lines expressing shRNAs targeting non-human genes (e.g. LacZ) were similarly generated and assayed (termed shControl). mRNA levels were robustly reduced by approximately 80 percent in the shSREBP2, shGGPS1 and shHMGCS1 cell lines relative to shControl lines (Figure 2A) as were the protein levels in the shSREBP2 and shHMGCS1 cell lines (Figure 2B). GGPS1 protein levels were not assessed due to the lack of specific antibodies.

**Figure 3:**
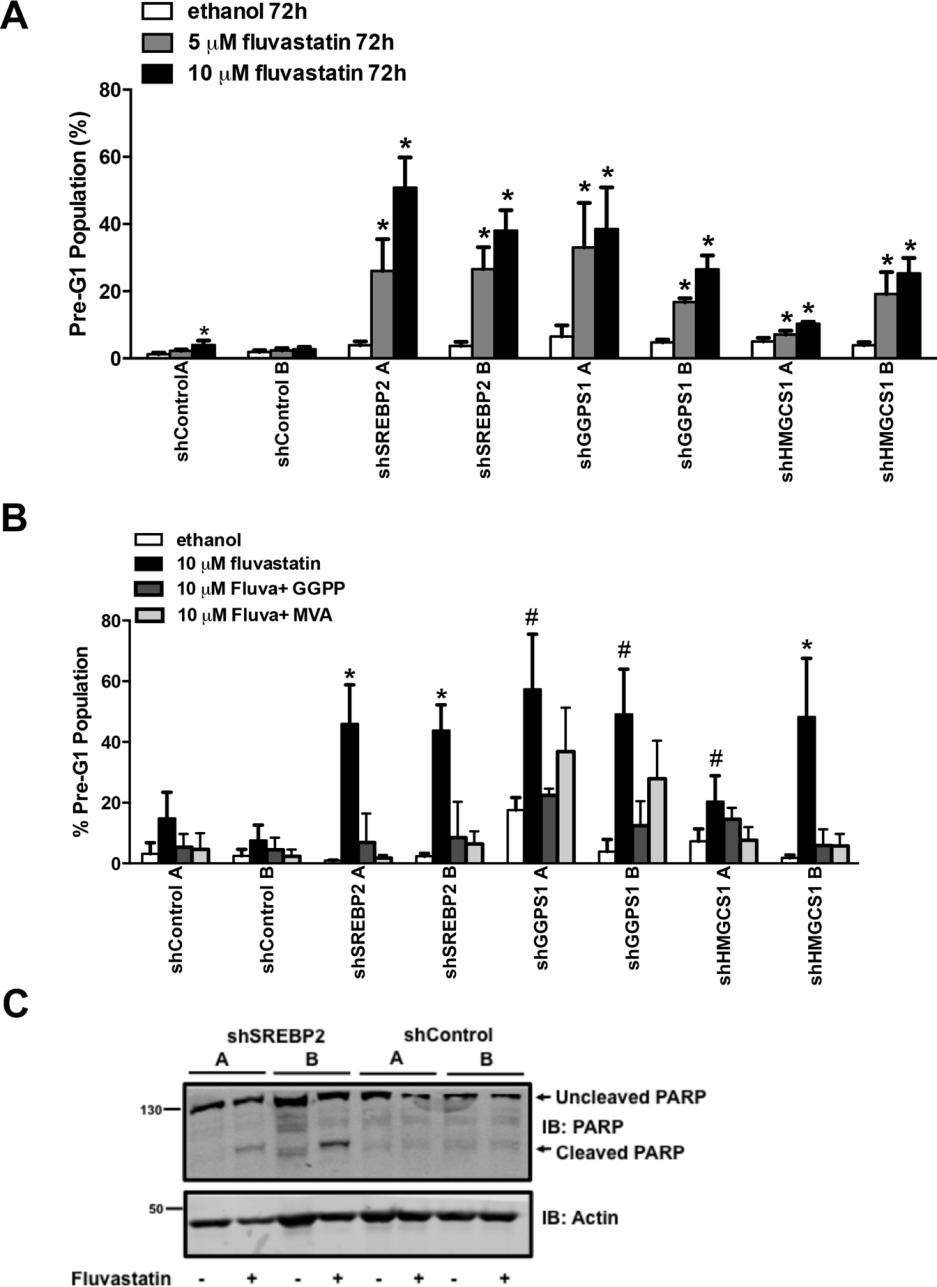
shRNAs targeting MVA pathway gene hits potentiate fluvastatin-induced apoptosis **A.** Exposure to fluvastatin (10 μM) for 72 hours of A549 cell lines stably expressing the indicated shRNA significantly induced apoptosis. **B.** Fluvastatin-induced apoptosis 72 hours post-treatment in the shGGPS1 cell lines was reversed by the concomitant addition of GGPP (10 μM) and not MVA (100 μM) but by both GGPP and MVA in shSREBP2 and shHMGCS1 cell lines. Bars represent the mean ± SD of at least three independent experiments * # *p* < 0.05 (*one-way ANOVA with Tukey-post test within each shA549 cell line comparing all groups, the fluvastatin-treated group being significantly different from the others, # the fluvastatin-treated group being significantly different than the ethanol and fluvastatin + GGPP groups). **C.** Protein lysates harvested from A549 cell lines treated with 10 μM of fluvastatin for 72 hours were immunoblotted and probed for PARP expression. Immunoblots are representative of at least three independent experiments.

The shSREBP2 and shGGPS1 cell lines were particularly sensitive to the anti-cancer effects of fluvastatin and this was reflected in the reduction of IC50 values relative to shControl lines (Figure 2C). When shSREBP2, shGGPS1 and shHMGCS1 A549 cell lines were treated with a sub-lethal dose of fluvastatin, we observed a dose-dependent induction of apoptosis as measured by cellular pre-G1 DNA content (Figure 3A). Apoptosis in the shControl fluvastatin-treated cells was minimal. The initial genome-wide results, combined with their independent validation confirms these gene products as targets whose knockdown in combination with fluvastatin administration increases tumor cell apoptosis.

The stable knockdown of SREBP2, GGPS1 and HMGCS1 can potentially perturb other pathways in addition to the MVA pathway. To verify that the apoptosis was MVA pathway dependent, we co-administered either MVA or GGPP with fluvastatin across our panel of shRNA cell lines. Fluvastatin-induced apoptosis was reversed by the co-administration of MVA or GGPP in the shSREBP2 and shHMGCS1 cell lines (Figure 3B) indicating that the apoptosis was dependent on MVA pathway inhibition and specifically dependent on the GGPP-producing arm of the MVA pathway. This result independently validates the functional importance of protein geranylgeranylation in the regulation of tumor cell death, and is consistent with previous reports showing that statin-induced apoptosis of several tumor types can be functionally blocked by the exogenous addition of MVA or GGPP [8, 25, 34]. Co-administration of GGPP (but not MVA) to fluvastatin-treated shGGPS1 cell lines blocked fluvastatin-induced apoptosis (Figure 3B). The absence of effect seen with co-administered MVA was expected because MVA production occurs several steps upstream of the GGPS1-catalyzed GGPP production. Providing additional evidence of apoptosis, treatment of shSREBP2 cell lines but not shControl A549 cells with fluvastatin induced PARP cleavage, which is an indicator of apoptosis downstream of caspase activation (Figure 3C). Taken together, knockdown of specific MVA pathway genes (SREBP2, GGPS1 and HMGCS1) can potentiate statin-induced apoptosis in A549 cells.

**Figure 4:**
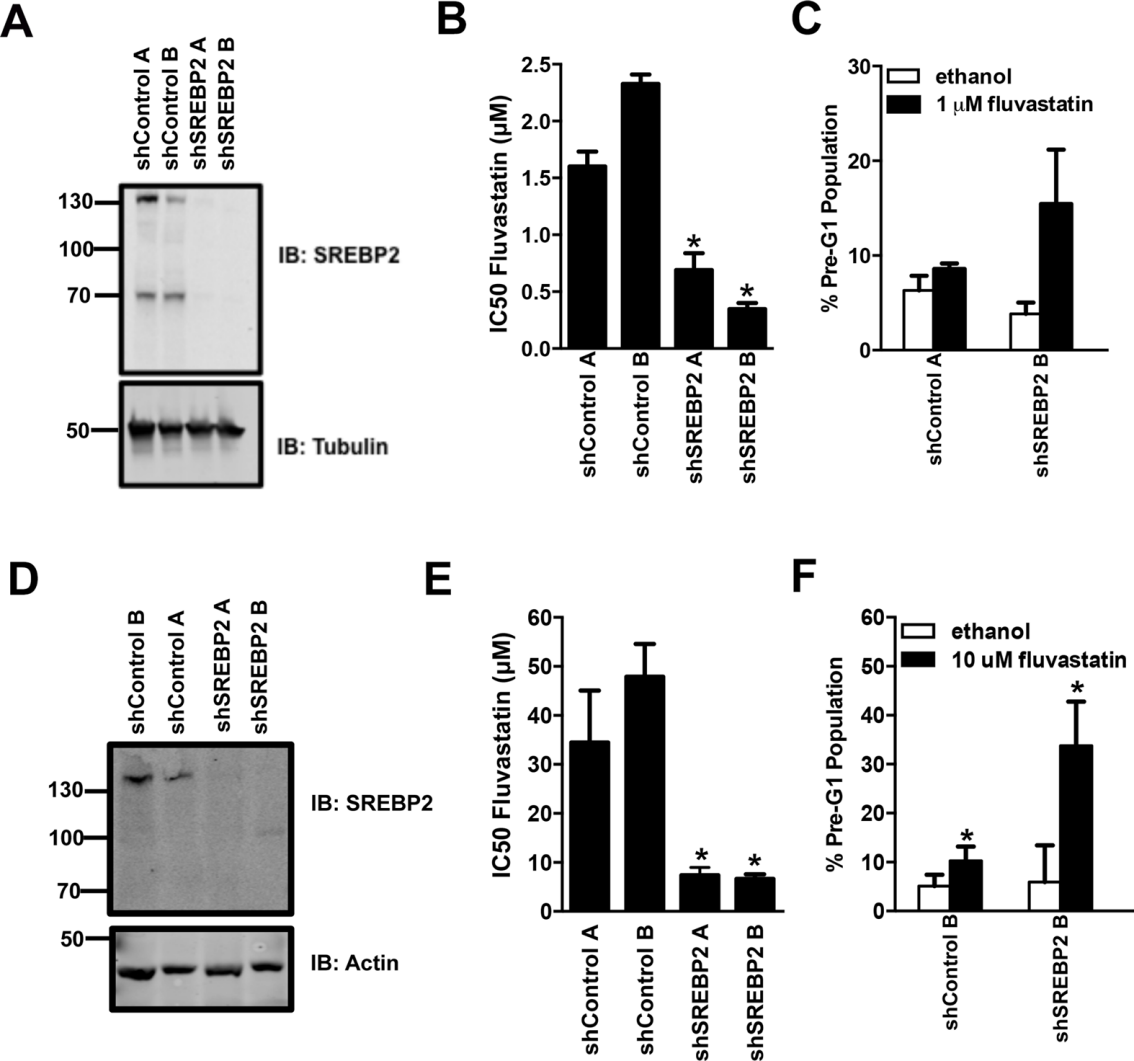
Stable knockdown of SREBP2 sensitizes breast cancer MDA-MB-231 and MCF7 cells to the pro-apoptotic and anti-proliferative effects of fluvastatin **A-F.** Protein lysates harvested from asynchronously growing MDA-MB-231 and MCF7 cells were immunoblotted for SREBP2 protein expression relative to cells transduced with the shControls (A and D respectively). Fluvastatin IC50 values in the MDA-MB-231 and MCF7 cell lines expressing the SREBP2 shRNAs were significantly lower when compared to the control cell lines (B and E respectively). Exposure of representative MDA-MB-231 and MCF7 cell lines to sub-lethal doses of fluvastatin caused a significant increase in apoptosis (C and F respectively). *p < 0.05 (*t*-test, unpaired, two-tailed, comparison made within each cell line). Immunoblots represent a minimum of two independent experiments.

**Figure 5:**
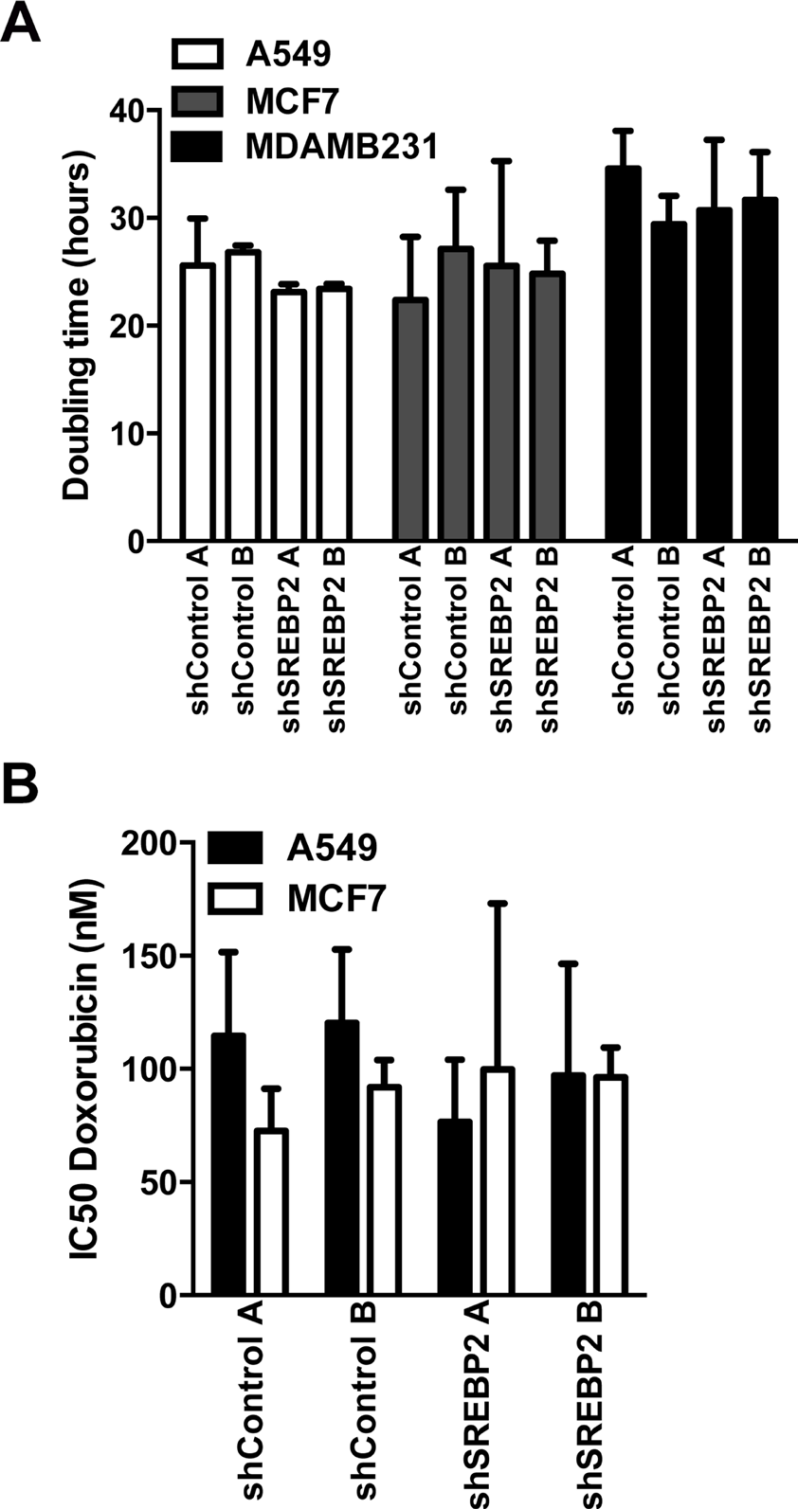
Knockdown of MVA pathway genes does not sensitize breast and lung cancer cells to the anti-proliferative effects of doxorubicin **A.** Proliferation as assessed by measuring the doubling time using the CyQuant cell proliferation assay kit was not significantly different between cell lines expressing the SREBP2 shRNAs and control cell lines in A549, MCF7 and MDA-MB-231 cells (one-way ANOVA with Tukey post test within each shA549 cell line comparing all groups). Bars represent the mean ± SD of at least three independent experiments. **B.** Doxorubicin IC50 values of the A549 and MCF7 cell lines expressing the SREBP2 shRNAs were not significantly different when compared to the cell lines expressing control shRNAs.

To evaluate the nature and generality of our results, we chose to further focus on the combination of statins with the knockdown of SREBP2, which not only decreased fluvastatin IC50 values but also triggered robust apoptosis. Furthermore, SREBP2 plays a central role in the MVA pathway as a master transcriptional activator of sterol responsive genes including HMGCS1 and HMGCR [12, 15]. To this end, we first tested whether SREBP2 knockdown also sensitized cells of another solid tumor type to fluvastatin-induced apoptosis. We chose breast cancer, as recent window-of-opportunity, pre-operative clinical trials showed that lipophilic statins (fluvastatin or atorvastatin) can impact tumor growth, suggesting statins may have a role in the management of this disease [19, 20]. The triple negative basal-like MDA-MB-231 and luminal MCF7 cells are relatively sensitive and insensitive to statin-induced apoptosis, respectively [35]. As previously observed with the A549 cells, MDA-MB-231 and MCF7 breast cancer cell lines stably expressing shRNAs targeting SREBP2 displayed increased fluvastatin sensitivity expressed as anti-proliferative and pro-apoptotic effects (Figure 4A–4F). SREBP2 knockdown alone in all cell lines tested did not perturb the rate of cell growth (assessed by doubling times) (Figure 5A). Furthermore, the sensitization effect was fluvastatin-specific as SREBP2 knockdown did not sensitize lung and breast cancer cells to the anti-cancer effects of doxorubicin, an anti-cancer drug with a distinct mechanism of action compared to fluvastatin (Figure 5B). Taken together, knockdown of SREBP2 specifically potentiated the anti-cancer effects of fluvastatin in lung and breast cancer cells.

**Figure 6:**
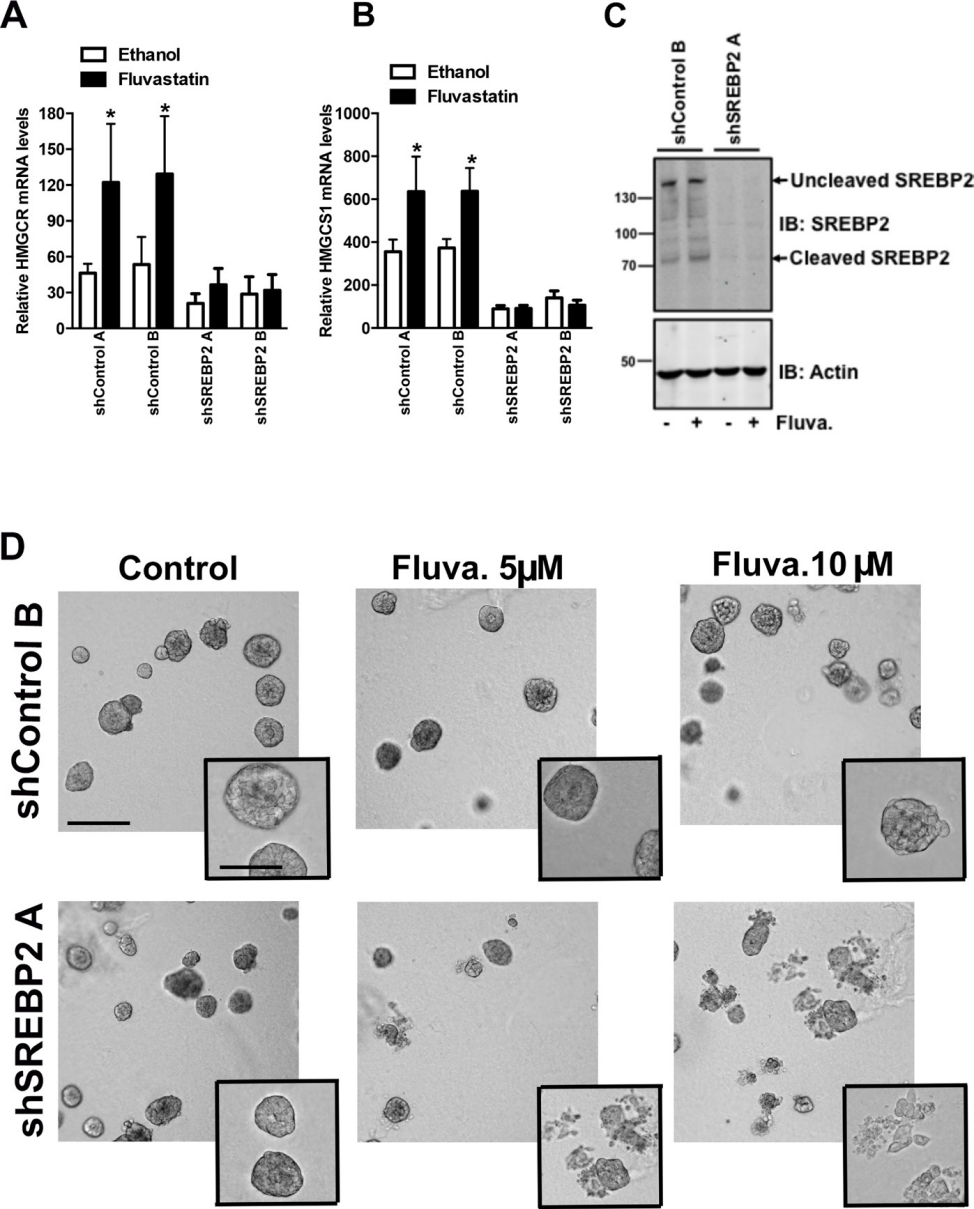
Knockdown of SREBP2 abrogates the sterol-feedback loop and impairs 3D growth upon fluvastatin treatment Cells were treated for 24 hours with 10 μM of fluvastatin or ethanol control and assessed for expression of **A.** HMGCR **B.** HMGCS1 relative to GAPDH in shA549 cell lines. Bars represent the mean ± SD of at least three independent experiments *p < 0.05 (*t*-test, unpaired, two-tailed, comparison made within each cell line). **C.** Protein lysates harvested from A549 cell lines treated with 10 μM of fluvastatin (Fluva.) for 24 hours were immunoblotted for SREBP2 expression. Immunoblots are representative of at least three independent experiments. **D.** Treatment with fluvastatin for 72 hours with the indicated fluvastatin concentrations disrupts A549 acini in shSREBP2 cell lines in 3D culture. Cells were imaged using an AxioObserver and are representative of three independent experiments. Scale bar represents 200 μm in main image and 100 μm in inset.

**Figure 7:**
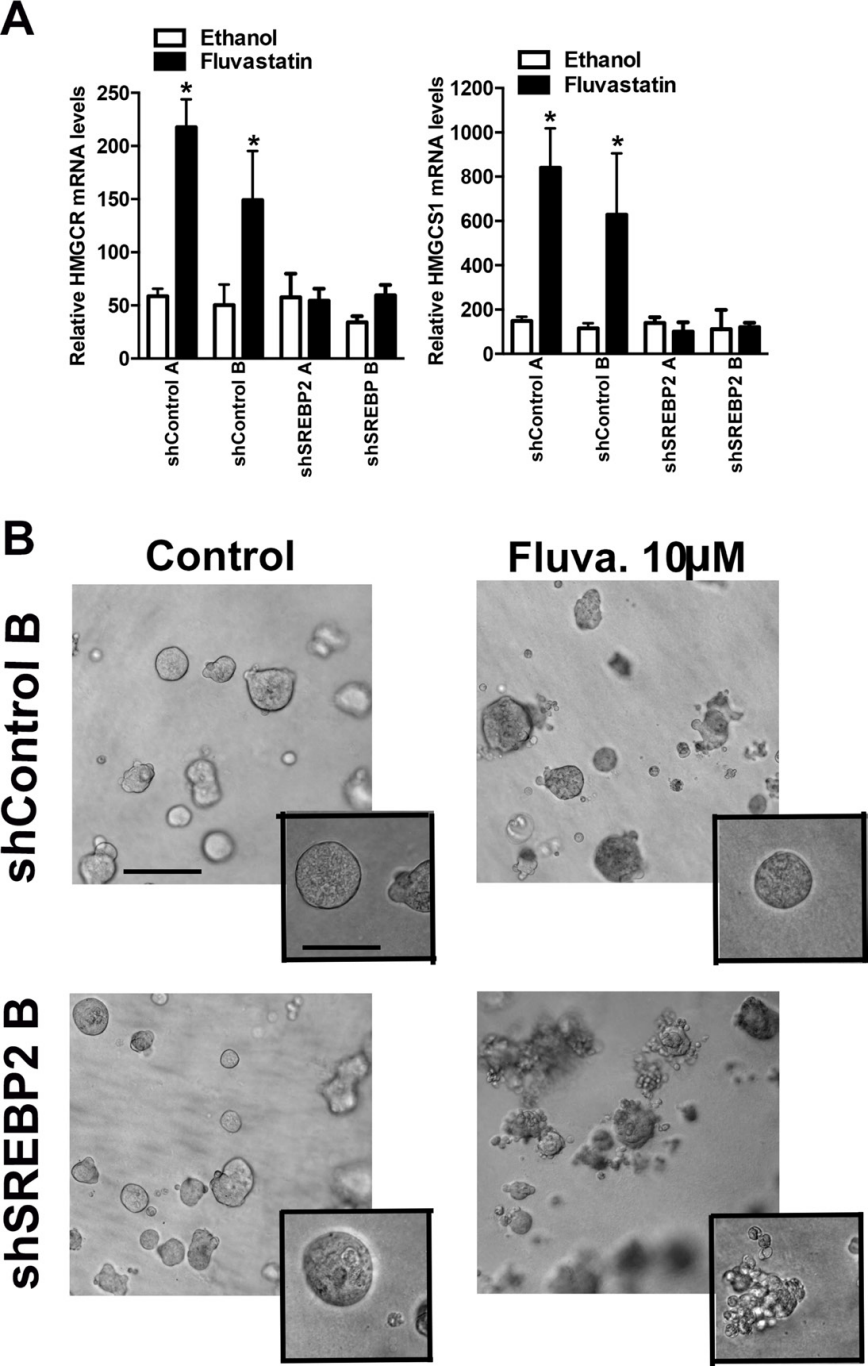
Knockdown of SREBP2 abrogates the sterol-feedback loop and impairs 3D growth of MCF7 cells upon fluvastatin treatment **A.** Cells were treated for 24 hours with 10 μM of fluvastatin or ethanol control and assessed for expression of HMGCR (A), HMGCS1 (B) relative to GAPDH in shMCF7 cell lines. Bars represent the mean ± SD of at least three independent experiments *p < 0.05 (*t*-test, unpaired, two-tailed, comparison made within each cell line). **B.** Treatment with fluvastatin for 72 hours with the indicated concentrations disrupts MCF7 acini in the shSREBP2 cell line in 3D culture but not in the shControl cell line. Images are representative of three independent experiments composed of three technical replicates. Scale bar represents 200 μm in main image and 100 μm in inset.

The sensitizing effects mediated by SREBP2 knockdown were completely reversed by co-administration of either MVA or GGPP. This functional rescue supports the model that SREBP2-mediated effects on the MVA pathway were responsible for the observed phenotype. Upon HMGCR inhibition and subsequent cholesterol depletion, SREBP2 initiates the transcription of sterol-responsive genes. This has been observed in many cancer cells and has been postulated to be an impediment to statin therapy [23, 36]. Upon fluvastatin treatment, HMGCR and HMGCS1 were significantly upregulated in the shControl A549 cells (Figure 6A and 6B). This was also observed in the MCF7 breast cancer cells (Figure 7A). The upregulation of HMGCR and HMGCS1 was accompanied by increased SREBP2 cleavage in the shControl lines (Figure 6C). The sterol-feedback response initiated upon HMGCR inhibition and mediated by SREBP2 cleavage was sufficient to blunt the anti-proliferative effects of fluvastatin but, as expected, was successfully abrogated with the knockdown of SREBP2 in shSREBP2 cell lines. In 3D culture growth, fluvastatin treatment disrupted the morphology and structure of the shSREBP2 A549 cells (Figure 6D) and shSREBP2 MCF7 cells [35, 37] (Figure 7B).

## DISCUSSION

By conducting a pooled, genome-wide shRNA screen, we have demonstrated the utility of targeting more than one member of the MVA pathway to further sensitize cells to fluvastatin. HMGCS1, GGPS1 and SREBP2 knockdown, in concert with statin-mediated HMGCR inhibition, can robustly enhance tumor cell apoptosis.

**Figure 8:**
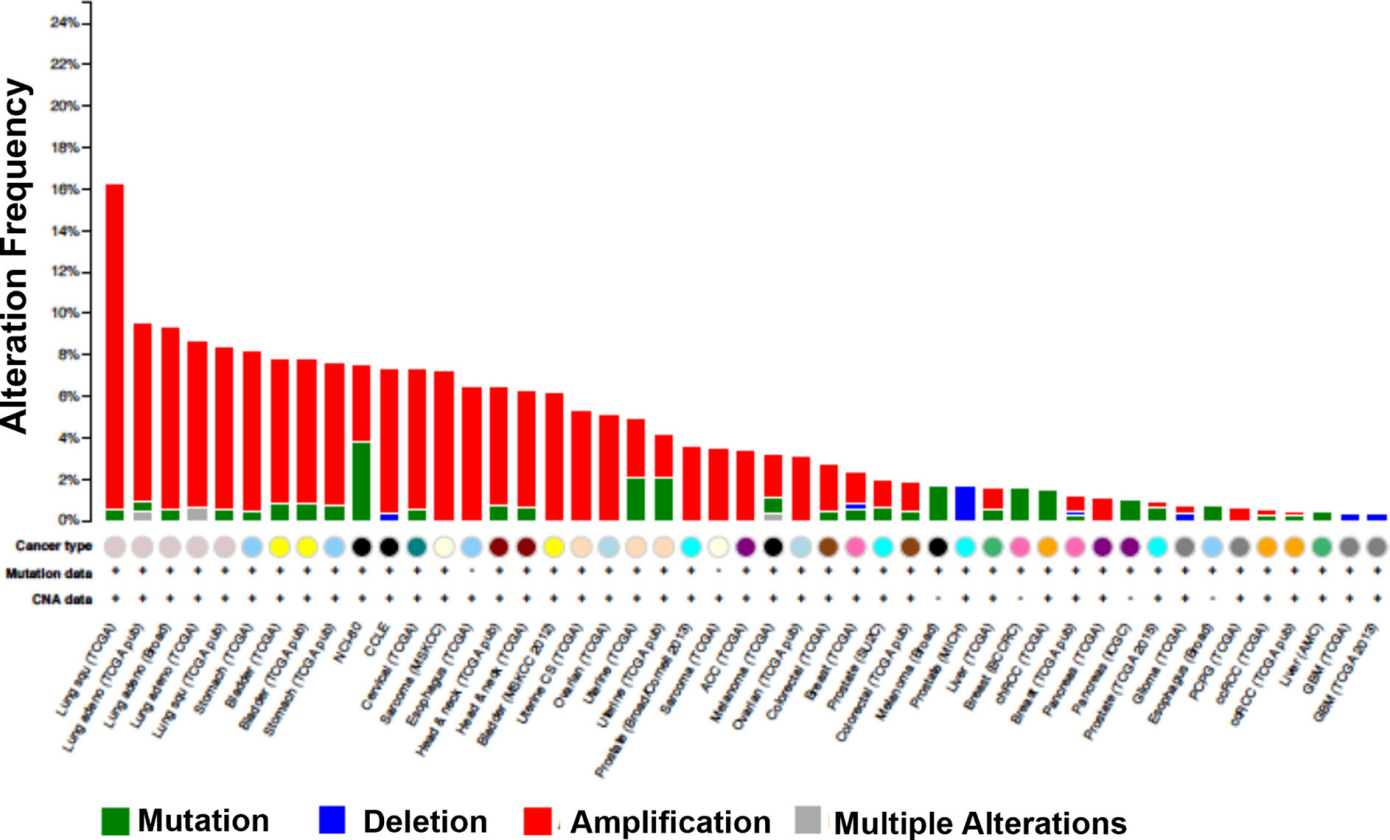
HMGCS1 is amplified in various cancers Analysis of cancer genomics datasets using cBioPortal demonstrating HMGCS1 genomic alterations across various tumor types.

Targeting HMGCS1 in combination with fluvastatin is attractive in tumor cells where HMGCS1 is robustly upregulated in response to statin treatment as was the case in the lung and breast cancer cell lines used in our study. In addition, analysis of cancer genomics datasets using cBioPortal [38, 39] shows that HMGCS1 can be amplified in cancers, including breast and lung (Figure 8). This suggests tumors with elevated basal HMGCS1 expression may be particularly responsive to treatment with fluvastatin and an inhibitor of HMGCS1.

Our finding that knocking down GGPS1 potentiates fluvastatin- mediated HMGCR inhibition is consistent with previous results from us and others that this arm of the MVA pathway is functionally critical for statin-induced apoptosis of tumor cells [25]. Moreover, recent results demonstrate dramatic synergy of pharmacological GGPS1 inhibitors was evident when combined with statins at inducing leukemia cell kill [40, 41]. GGPS1 is an emerging novel therapeutic target that provides an alternative to the existing, but not highly efficacious single agent farnesyl and gernaylgeranyltransferase (GGTase) inhibitors designed to target isoprenoid biosynthesis [42, 43]. This inspires a novel model by which the statin-triggered feedback loop would restore the MVA end-products, but in the presence of GGPS1 inhibitors, this lethal vulnerability would not be rescued leading to tumor cell death. As HMGCR is the rate-limiting enzyme of the MVA pathway, its inhibition should lead to a depletion of all products downstream of MVA. Unexpectedly, we observed a strong fluvastatin potentiation upon knockdown of specific genes in the pathway (GGPS1 and HMGCS1) and further study is required to delineate the balance between HMGCR inhibition and the counteracting effects of the sterol-feedback loop.

In normal cells, the *de novo* lipid synthesis pathways are under tight physiological control by the sterol response element binding protein (SREBP) family of transcription factors, SREBP1 and SREBP2. Although the physiological role of SREBP2 in maintaining cholesterol homeostasis is well defined, the importance of this basic helix-loop-helix leucine zipper transcription factor in cancer cells and oncogenesis remains relatively unexplored unlike that of SREBP1, which is highly activated in various cancers and its activation is linked with the PI3K/Akt/mTOR signalling axis [44–46]. SREBP2 is over-expressed during progression in primary prostate cancer cells [46], and combined SREBP2 and SREBP1 knockdown in cancer cell lines caused endoplasmic reticulum stress and induction of apoptosis in lipoprotein-depleted conditions [47]. We found that SREBP2 knockdown did not affect cell growth in lung and breast cancer cells, and that differences between the shSREBP2 and shControl cell lines were only apparent following fluvastatin exposure. Although no specific SREBP2 inhibitors are available, we recently identified another drug, dipyridamole, that effectively blocked SREBP2 cleavage and synergized with statins to induce apoptosis in haematological malignancies indicating that SREBP2 is a novel promising therapeutic target [36].

Targeting SREBP2 effectively suppresses the restorative feedback loop triggered by statin treatment, widening the therapeutic window to include tumors otherwise unresponsive to statins. Therefore, SREBP2 is an especially attractive and novel target to have emerged from the screen. Statins are safe, FDA-approved and off-patent, making them cost-effective anti-cancer agents that may be explored in future combination chemotherapies, and here, we have identified three novel targets augmenting their efficacy. In addition, given the inherent homeostatic feedback loops that are built into the metabolic circuitry of a cell, the concept of blocking feedback loops that blunt the efficacy of initial pathway inhibition are important as more anti-cancer agents targeting abnormal tumor metabolism are developed.

## MATERIALS AND METHODS

### Cell culture

Cell lines were maintained in Dulbecco's Modified Eagle Medium was supplemented with 10% fetal bovine serum (GIBCO) and penicillin-streptomycin.

### shRNA screen, lentivirus and shRNA cell line generation

A549 cell line, stably transduced with the RNAi Consortium (TRC1) shRNA library [27] was treated with fluvastatin (US Biologicals) over 12 days. Genomic DNA (gDNA) was amplified and shRNA populations hybridized onto custom Affymetrix Gene Modulation Array Platform (GMAP) arrays. Differences between shRNA abundances over 12 days were assessed using the Bayes Factor [29]. For generation of stably expressing shRNA cell lines, DNA from bacterial glycerol stocks of shRNAs from the TRC1 library was amplified using *E.coli* and purified (Qiagen Plasmid Maxiprep kit). Lentiviral particles were generated by calcium phosphate transfection of sub-confluent (50–60%) HEK293TV cells with 10 μg of TRC pLKO.1 puro shRNA construct, 5 μg each of pMDG1.vsvg, pRSV-Rev and pMDLg/pRRE constructs. Lentiviral particles were collected 24 and 48 hours later, filtered though a 0.45 μm filter and stored at −80°C. Parental cell lines were infected with lentiviral particles containing the indicated shRNAs and puromycin selected (48 hrs, 2 μg/ml of puromycin).

### Immunoblotting

For SREBP2, HMGCS1 and PARP protein detection, cells were lysed using boiling SDS lysis buffer (1.1% SDS, 11% glycerol, 0.1 M Tris pH 6.8) with 10% β-mercaptoethanol). Blots were probed with anti-SREBP2 (BD Pharmingen), anti-HMGCS1 (Santa Cruz Biotechnology), anti-PARP (Cell Signaling) anti-tubulin (Santa Cruz Biotechnology) and anti-actin (Sigma) and detected using the Odyssey infrared imaging system (LI-COR Biosciences).

### MTT, fixed propidium iodide (PI) and CyQuant proliferation assays

MTT (Sigma-Aldrich) assays were done as previously described [23, 36]. Briefly, 1.5–2.0 × 10^4^ cells/ml were plated in 96-well plates and after 24 hours, treated with a range of fluvastatin and doxorubicin concentrations for 72 hours. Half-maximal inhibitory concentrations (IC50) values were computed from dose-response curves using Prism (v5.0, GraphPad Software). For fixed PI assays, cells were fixed in 70% ethanol, treated with DNAse-free RNAse and stained with PI (Sigma-Aldrich). Cells were analyzed for the dying pre-G1 population by flow cytometry. Cell proliferation assays were carried out as per manufacturer's protocol (Life Technologies).

### Real-time PCR

RNA was harvested from asynchronously growing cells and isolated using Trizol (Invitrogen). mRNA knockdown of SREBP2 (Hs01081784_m1), GGPS1 (Hs00191442_m1) and HMGCS1 (HS00266810_m1) relative to GAPDH (Hs99999905_m1) were assessed using TaqMan Gene Expression Assays using TaqMan master mix (Applied Biosystems). HMGCR mRNA levels were assayed as previously described [23].

### 3D Morphogenesis

Performed as previously described [35].

## SUPPLEMENTARY TABLE




