# Supplementary material for: Genome-wide RNAi analysis reveals that simultaneous inhibition of specific mevalonate pathway genes potentiates tumor cell death
Source: Oncotarget. 2015 Aug 22;6(29):26909–21. doi: 10.18632/oncotarget.4817 (PMC4694962; doi:10.18632/oncotarget.4817)
Supplement: Supplementary file 1 [file oncotarget-06-26909-s001.pdf]

SUPPLEMENTARY TABLE

Supplementary Table S1: A complete list of all the under-represented shRNAs in the fluvastatin-treated A549 cells compared to ethanol control-treated cells.
